# An Umbrella Review of Effectiveness of Intravenous Ketamine in Treatment-Resistant Depression

**DOI:** 10.1192/j.eurpsy.2024.222

**Published:** 2024-08-27

**Authors:** A. M. Klassen, C. Baten, J. H. Shepherd, G. Zamora, E. Johnson-Venegas, S. S. Madugula, E. Woo, J. A. Miller, M. D. Sacchet, D. W. Hedges, C. H. Miller

**Affiliations:** ^1^Department of Psychology, California State University, Fresno, Fresno; ^2^School of Medicine, Stanford University, Stanford; ^3^Department of Psychology, Palo Alto University, Palo Alto; ^4^Department of Psychiatry, Massachusetts General Hospital, Harvard Medical School, Boston; ^5^Department of Psychology, Brigham Young University, Provo, Provo, United States

## Abstract

**Introduction:**

Major depressive disorder (MDD) is a tremendous global disease burden and the leading cause of disability worldwide. Unfortunately, individuals diagnosed with MDD typically experience a delayed response to traditional antidepressants and many do not adequately respond to pharmacotherapy, even after multiple trials. The critical need for novel antidepressant treatments has led to a recent resurgence in the clinical application of psychedelics, and intravenous ketamine, which has been investigated as a rapid-acting treatment for treatment resistant depression (TRD) as well acute suicidal ideation and behavior. However, variations in the type and quality of experimental design as well as a range of treatment outcomes in clinical trials of ketamine make interpretation of this large body of literature challenging.

**Objectives:**

This umbrella review aims to advance our understanding of the effectiveness of intravenous ketamine as a pharmacotherapy for TRD by providing a systematic, quantitative, large-scale synthesis of the empirical literature.

**Methods:**

We performed a comprehensive PubMed search for peer-reviewed meta-analyses of primary studies of intravenous ketamine used in the treatment of TRD. Meta-analysis and primary studies were then screened by two independent coding teams according to pre-established inclusion criteria as well as PRISMA and METRICS guidelines. We then employed metaumbrella, a statistical package developed in R, to perform effect size calculations and conversions as well as statistical tests.

**Results:**

In a large-scale analysis of 1,182 participants across 51 primary studies, repeated-dose administration of intravenous ketamine demonstrated statistically significant effects (p<0.05) compared to placebo-controlled as well as other experimental conditions in patients with TRD, as measured by standardized clinician-administered and self-report depression symptom severity scales.

**Conclusions:**

This study provides large-scale, quantitative support for the effectiveness of intravenous, repeated-dose ketamine as a therapy for TRD and a report of the relative effectiveness of several treatment parameters across a large and rapidly growing literature. Future investigations should use similar analytic tools to examine evidence-stratified conditions and the comparative effectiveness of other routes of administration and treatment schedules as well as the moderating influence of other clinical and demographic variables on the effectiveness of ketamine on TRD and suicidal ideation and behavior.

**Disclosure of Interest:**

None Declared

